# Dehydrated amniotic membrane patch for the treatment of an ocular event secondary to mirvetuximab soravtansine-gynx (MIRV) therapy

**DOI:** 10.1016/j.gore.2024.101547

**Published:** 2024-11-19

**Authors:** Brittany File, Anjali Hari, Ling Bei

**Affiliations:** a Department of Obstetrics and Gynecology, University of California, Irvine Medical Center, Orange, CA, USA; b Department of Obstetrics and Gynecology, Division of Gynecologic Oncology, University of California, Irvine Medical Center, Orange, CA, USA; c Department of Ophthalmology, Long Beach Memorial Medical Center, Long Beach, CA, USA

**Keywords:** Amniotic membrane patch, Elahere therapy, Mirvetuximab soravtansine (MIRV), Ocular event, Case report

## Abstract

• Mirvetuximab soravtansine (MIRV) is used in the treatment of FRα positive, platinum-resistant epithelial ovarian cancer.
• MIRV is associated with ocular events, leading to dose delays, dose reductions, and discontinuations of chemotherapy.
• Mitigation strategies to prevent treatment disruption have historically relied on corticosteroid and lubricating drops.
• Dehydrated amniotic membrane patch to treat MIRV related ocular events is a novel therapeutic option.

## 1 Introduction

Mirvetuximab soravtansine (MIRV) is an antibody-drug conjugate (ADC) composed of an FRα binding antibody approved by the FDA in November of 2022 for the treatment of FRα positive, platinum-resistant epithelial ovarian cancer (Matulonis et al., 2023).

While overall well tolerated, one of the most common treatment-related adverse events is ocular events (Moore et al., 2022). Mitigation strategies with primary prophylaxis utilizing daily corticosteroid eye drops and lubricating eye drops have been implemented to reduce adverse events (Hendershot et al., 2023, Matulonis et al., 2019). Descriptions of topical steroids and lubricants for ocular events related to MIRV toxicity continue to be the sole treatment option described in the literature (Corbelli et al., 2019, Kunkler et al., 2019). As a result of ocular events, patients are often required to undergo dose delays and reductions until symptom improvement or resolution (Moore et al., 2022, Eaton et al., 2015).

Amniotic membrane patches have demonstrated remarkable potential to treat corneal and epithelial damage, dry eyes, keratopathy and other ocular conditions (Walkden, 2020, Meller et al., 2011). We describe a patient with worsening eye dryness after treatment with topical steroids for an ocular event related to MIRV being treated with topical stem cell treatment membrane with resolution of symptoms.

## 2 Case

A 61-year-old patient initially presented with bilateral adnexal masses, peritoneal carcinomatosis and underwent primary cytoreductive surgery with no gross residual disease in November of 2017 found to have International Federation of Gynecology and Obstetrics (FIGO) Stage IIIC high grade serous fallopian tube carcinoma. She unfortunately progressed through multiple lines of chemotherapy and in July of 2022 her CA125 was 35 with scans demonstrating interval disease progression.

Her tumor was tested for FRα expression and was found to be positive with 80 % expression.

She was seen by an ophthalmologist in January of 2023 and had a baseline eye exam which cleared her for use of MIRV. At this exam, her vision without correction was 20/30 in both eyes and no signs of corneal epithelial keratitis were noted. Her CA125 at that time was 446. She was given a prescription for prednisolone drops as well as preservative free lubricating drops and underwent Cycle 1 on February 6th with the plan for 6 mg/kg adjusted ideal body weight (AIBW) every 3 weeks for 9 cycles. She was evaluated by the ophthalmologist on C1D21 with minimal keratitis noted and stable visual acuity of 20/40 in both eyes.

She underwent Cycle 2 and had a downtrending CA125 to 364. On C2D9 she presented with new complaint of bilateral blurry vision and dry eyes. She reported excellent compliance with her eye drops. She saw her ophthalmologist the same week and her exam demonstrated new diffuse corneal epithelial keratitis with subsequent drop in vision to 20/150 in both eyes. In the office, bilateral lower eyelid silicone eyelid punctal plugs were placed with the goal to increase ocular lubrication by slowing the outflow of the patient’s tears. The patient was also prescribed lubricating eye ointment for nighttime use and artificial tear gel drops to be used every 2 h while awake. One week later, her exam demonstrated moderate improvement in keratitis, though persistence in blurry vision.

Given these changes, she underwent dose reduction prior to Cycle 3 with plan for 5 mg/kg AIBW starting with Cycle 3. She then completed Cycle 3 at the end of March and reported improved blurry vision. Her eye exam on C3D3 showed improved visual acuity to 20/80 in both eyes; however, this was still significantly worse than her baseline. She continued to have down trending CA125 to 90 at this time. On C3D22 the patient reported persistently extremely dry eyes and blurred vision. She received Cycle 4 the same week with persistent blurry vision, though overall improved with dose reduction and approved by ophthalmology to continue. Her CA125 had downtrended to 42. She continued reporting compliance in eye drops. She met with her ophthalmologist on C4D3 and was instructed to stop using the steroid eye drops until her vision improved secondary to confluent superficial keratitis with persistent dry eyes. Generally, it is believed that use of high potency topical steroids on the eye can delay wound healing in the context of significant epithelial keratitis. On C4D16, her vision had improved to 20/60 right and 20/40 in her left eye.

In conjunction with ophthalmology, it was decided to delay Cycle 5 for two weeks. Given the persistent and dramatic worsening of her keratitis which would exacerbate about one week after each cycle, the decision was made to place an amniotic membrane to encourage corneal limbal stem cell growth to rehabilitate the cornea surface. At the end of April on C4D19, her ophthalmologist placed a dehydrated amniotic membrane (AcellFx) in the left eye.

Patient underwent Cycle 5 at the beginning of May after being cleared by ophthalmology with continued improvement in CA125 to 30. On C5D12 she did not demonstrate corneal decompensation as in previous cycles and her vision stabilized at 20/40 in the left eye which had membrane placement. A dehydrated amniotic membrane (AcellFx) was placed in her right eye on C5D12.

She reported improvement in her overall vision. She underwent Cycle 6 at the end of May without complaint or ocular symptoms – she continued to meet with ophthalmologist for stem cell treatments including a replacement membrane in her left eye approximately 4 weeks after initial placement, and again approximately 3–4 weeks later. She received replacement membranes in the right eye two additional times. She underwent Cycle 7 in mid-June with CA125 at this time increased to 38. She then completed Cycle 8 in mid-July with another increase in her CA125 to 88. She was diagnosed with COVID in August and underwent treatment break and during this time had a CA125 of 182.

She completed a PET/CT at the beginning of September 2023 (C8D59) with interval smaller FDG avid and no FDG avid hepatic lesions, resolution of her cardiophrenic lesions; however, noted persistent right pelvic sidewall nodes. Her CT scan completed on the same date demonstrated interval increase of hepatic flexure serosal mass and questionable left pelvic nodule mass with more apparent left hepatic subcapsular lesion concerning for progression. Given discrepancy in imaging, decision was made to present case at tumor board where it was decided to proceed with radiation oncology consult and Cycle 9 given travel holiday treatment break.

She met with radiation oncology at the beginning of October with the plan to treat her only site of FDG avid disease with stereotatic ablative radiotherapy with 30 Gray in 5 fractions to the FDG avid node and 25 Gray in 5 fractions to the lymph node immediately superior and inferior to the FDG avid lymph node for microscopic disease. Her CA125 on C8D85 had increased to 210. She underwent Cycle 9 in the middle of October and radiation the same week. Her CA125 on C9D8 was 290. Given prior discrepancy in imaging and rising CA125, decision was made to discontinue MIRV and screen patient for clinical trials.

Ophthalmology exam on C9D24 showed rapidly growing cataracts in both eyes. Her vision in her right eye was 20/60 and her left eye had now deteriorated to 20/100 despite no keratitis noted in either eye. On C9D31 her CA125 had decreased to 206, but given significant worsening of cataracts and vision, MIRV was no longer considered. On C9D61 her CA125 had increased to 402. She underwent successful cataract surgery in the left eye in mid-January 2024 with vision recovery to 20/25 after surgery.

Her CA125 On C9D96 was 601. She was subsequently enrolled in a clinical trial where she received 2 cycles with noted progression and was transitioned to Carboplatin/Doxorubicin in May of 2024 and received 4 cycles prior to being hospitalized with further progression and multifocal malignant small bowel obstruction. She was ultimately transitioned to comfort care and died in September of 2024. Her last documented CA125 in August was 2,331.

## 3 Discussion

MIRV gained its approval in November of 2022 as a result of the SORAYA study – a single arm trial of 106 patients with FRα – high, platinum-resistant ovarian cancer who had received 1 to 3 prior therapies, including bevacizumab. The trial results demonstrated an objective response rate of 32.4 % (95 % CI, 23.6 to 42.2), with 5 complete and 29 partial responses of the 105 patients evaluable for efficacy (Matulonis et al., 2023).

In the safety evaluation of MIRV, they cited all-grade and grade 3–4 treatment-related adverse events, respectively of blurred vision (41 % and 6 %), keratopathy (29 % and 9 %), and nausea (29 % and 0 %). These events led to 33 % of patients having dose delays, 20 % having reductions in dosing, and 9 % having therapy discontinued entirely (Matulonis et al., 2023).

The ocular events of MIRV are thought to be secondary to “off-target” effects related to the disruption mitosis within the corneal epithelium causing microcystic-like epithelial changes (Canestraro et al., 2022, Nguyen et al., 2023). In a pooled data safety analysis of MIRV, ocular events were the most commonly reported adverse event in patients treated with MIRV with the most common being vision impairment (49 %), keratopathy (36 %), and dry eyes (26 %) (Moore et al., 2022, Wang et al., 2024).

Due to the ability of the corneal epithelium to regenerate via in situ limbal epithelial stem cells, the ocular side effects of MIRV are not permanent (Tseng, 1989). In fact, in the safety summary completed for MIRV, 57 % of ocular events related to MIRV resolved without dose modification and improved within 14 days (Moore et al., 2022). Discontinuation of MIRV related to ocular events occurred in < 1 % of patients in the pooled data safety analysis. They found 12 % of total patients required a dose reduction, 23 % of which were in the ocular adverse event group. No severe or permanent ocular sequela were noted in the safety analysis, and for all patients’ ocular effects resolved to Grade 1 or Grade 0 (Moore et al., 2022).

Mitigation strategies have been developed in an attempt to reduce the frequency and severity of ocular events for patients being treated with MIRV including dosing strategies, daily use of lubricating eye drops, avoiding use of contact lenses, eyelid cleaning protocols, warm compresses, UV eye protection (Hendershot et al., 2023, Matulonis et al., 2019, Wang et al., 2024, Moore et al., 2017). These efforts were found to be successful in reducing the frequency and grade of ocular adverse events (Matulonis et al., 2019, Moore et al., 2017). The use of daily corticosteroid eye drops does not reduce the total number of ocular events; however, it has been adopted as part of the standard protocol due to evidence that patients require fewer dose reductions (Matulonis et al., 2019). The above strategies are the standard of practice to prevent ocular adverse effects in patients prescribed MIRV (Hendershot et al., 2023).

Human amniotic membrane has been shown to provide a substantial benefit in treating certain types of conjunctival and corneal disease and is an FDA-approved class II medical device. Amniotic membrane is an avascular fetal membrane that lies deep to the chorion and is harvested in a sterile environment from placental tissue obtained during elective cesarean sections. Donors are screened for transmissible diseases, and the amniotic membrane is further treated with broad-spectrum antibiotics immediately after collection (Jirsova and Jones, 2017).

Amniotic membrane is made up of three layers: epithelium, basement membrane, and stroma. The intrinsic trophic components including epidermal growth factor (EGF), keratocyte growth factor, transforming growth factor (TGF) β1 promote cell growth and support damage tissue by facilitating migration of epithelial cells, promoting cell differentiation amongst other effects (Walkden, 2020, Meller et al., 2011). Several types of collagens make up the basement membrane, including type VII collagen, which is also present in the conjunctival and corneal basement membranes. Dehydrated amniotic membrane is preserved using vacuum with low temperature heat to retain devitalized cellular components (Jirsova and Jones, 2017).

The use of amniotic membranes is widespread to treat a wide spectrum of inflammatory (eg. Dry eye disease, non-healing burn injuries) and immune-related ophthalmic diseases (stevens Johnson syndrome, non-healing corneal ulcers) (Dekaris et al., 2010, Kheirkhah et al., 2008, Pachigolla et al., 2009).

AcellFx is a human amniotic membrane processed with removal of the epithelium and chorion layers producing an acellular membrane, which is thought to lower the risk of immune response. It is composed of a variety of bio factors including collagen, fibronectin, laminin, and proteoglycan (Théa Pharma Inc, 2022). Early loss of membrane may occur if it is not anchored securely. Keratitis due to contamination of the membrane may also occur. On the whole, there are no major complications known to occur with amniotic membrane graft. Rarely, HIV, hepatitis B, and hepatitis C may be transmitted by amniotic membrane graft. Risks and benefits of placement are discussed extensively with the patient in the office prior to placement of the membrane (Srinivasan et al., 2007).

To our knowledge, use of amnion transplantation for the treatment of ocular adverse events secondary to chemotherapy has not yet been described. This promising new approach for management of ocular toxicity may reduce the need for dose delays, reductions and discontinuations and ultimately allow more patients to benefit from MIRV. Given limited existing data, future studies are warranted to further investigate this newly described therapeutic approach.

The addition of therapeutic options for the treatment of ocular adverse events secondary to chemotherapy provides immense possibility in the prevention of delays, dose reductions, and discontinuation of cancer treatment options.

Study approval statement: This study protocol was reviewed and approved by the University of California Institutional Review Board.

Consent to publish statement: Written informed consent was obtained from the patient for publication of this case report. A copy of the written consent is available for review by the Editor-in-Chief of this journal on request.

## Funding Sources

The study was not supported by any sponsor or funder.

## Author Contributions

BF: Conceptualized topic, investigation of topic, and wrote the manuscript.

AH: Conceptualized topic, provided senior guidance and supervision.

LB: Contributed information for case presentation.

All authors approved the final version of the manuscript.

## CRediT authorship contribution statement

**Brittany File:** Writing – original draft. **Anjali Hari:** Supervision. **Ling Bei:** Writing – original draft.

## Declaration of competing interest

The authors declare that they have no known competing financial interests or personal relationships that could have appeared to influence the work reported in this paper.
